# Potential of Dual and Multi Energy XRT and CT Analyses on Iron Formations

**DOI:** 10.3390/s21072455

**Published:** 2021-04-02

**Authors:** Christine Bauer, Rebecca Wagner, Beate Orberger, Markus Firsching, Alexander Ennen, Carlos Garcia Pina, Christiane Wagner, Maryam Honarmand, Ghasem Nabatian, Iman Monsef

**Affiliations:** 1 Development Center X-ray Technology EZRT, Fraunhofer Institute for Integrated Circuits IIS, Flugplatzstr. 75, 90768 Fürth, Germany; christine.bauer@iis.fraunhofer.de (C.B.); rebecca.wagner@iis.fraunhofer.de (R.W.); alexander.ennen@iis.fraunhofer.de (A.E.); 2 Géosciences Paris Sud (GEOPS), Bâtiment 504, Rue du Belvédère Campus Universitaire d’Orsay, 91405 Orsay CEDEX, France; beate.orberger@universite-paris-saclay.fr; 3 Catura Geoprojects, 2 rue Marie Davy, 75014 Paris, France; 4 DMT GmbH & Co. KG, Am TÜV 1, 45307 Essen, Germany; Carlos.GarciaPina@dmt-group.com; 5 CNRS-INSU, Sorbonne Université, ISTeP UMR 7193,F-75005 Paris, France; christiane.wagner_raffin@sorbonne-universite.fr; 6 Department of Earth Sciences, Institute for Advanced Studies in Basic Sciences (IASBS), Zanjan 45137-66731, Iran; m.honarmand@iasbs.ac.ir (M.H.); iman.monsef@iasbs.ac.ir (I.M.); 7 Department of Geology, Faculty of Science, University of Zanjan, University Blvd., Zanjan 45371-38791, Iran; gh.nabatian@znu.ac.ir

**Keywords:** X-ray, computed tomography, X-ray transmission, banded iron ore, nodular iron ore, quartz content, iron ore processing, dual energy, basis material decomposition, multi energy

## Abstract

Dual and multi energy X-ray transmission imaging (DE-/ME-XRT) are powerful tools to acquire quantitative material characteristics of diverse samples without destruction. As those X-ray imaging techniques are based on the projection onto the imaging plane, only two-dimensional data can be obtained. To acquire three-dimensional information and a complete examination on topology and spatial trends of materials, computed tomography (CT) can be used. In combination, these methods may offer a robust non-destructive testing technique for research and industrial applications. For example, the iron ore mining and processing industry requires the ratio of economic iron minerals to siliceous waste material for resource and reserve estimations, and for efficient sorting prior to beneficiation, to avoid equipment destruction due to highly abrasive quartz. While XRT provides information concerning the thickness, areal density and mass fraction of iron and the respective background material, CT may deliver size, distribution and orientation of internal structures. Our study shows that the data provided by XRT and CT is reliable and, together with data processing, can be successfully applied for distinguishing iron oxide rich parts from waste. Furthermore, heavy element bearing minerals such as baryte, uraninite, galena and monazite can be detected.

## 1. Introduction

X-ray transmission (XRT) can reveal information on internal structures of samples or different sample compositions. However, this information is limited as XRT results in an X-ray projection that reveals only two-dimensional (2D) features while averaging over the third dimension. Consequently, materials that are thick and low absorbing cannot be distinguished from those that are thin and strongly absorbing. Still, this technology was tested for nanosized and nanostructured CuO materials [1] and recently for sensor-based sorting of mine material in the project X-mine (funded by the European Union’s Horizon 2020 research and innovation program) [2].

Dual energy (DE) or multi energy (ME) XRT use the energy dependence of X-ray attenuation to obtain more material information than would be possible from standard radiographs. These techniques allow acquiring quantitative material characteristics like the effective atomic number or the areal density [3,4]. Today, DE imaging is widely known for the use in medical and security applications but can also show surprisingly good results in the equally promising sectors of non-destructive testing (NDT) and sorting [5,6].

The idea of X-ray computed tomography (CT) was first mentioned in a Soviet-Russian publication of Korenblum et al. in 1958 [7] but found lesser attention due to the language barrier than the well-known publication of Hounsfield in 1973 [8]. In both early cases, the method was developed for the use in medical imaging but today CT is also applied in other fields like earth sciences, and exploration and mining [9,10,11]. It is a non-destructive technique that allows three-dimensional (3D) visualization of internal structures of rock samples. These structures are mainly defined by variations in density and atomic composition of the compounds.

In mining, deciphering texture of ore minerals and parameter definition for mineral or metallurgical processing are crucial. CT was tested and successfully applied for Ni–Cu–platinum group elements (PGE) [12] and copper–gold mineralization [13]. Furthermore, it was applied to 3D interconnected fracture systems [14], and internal structures of hydrothermally altered submarine volcanic rocks to investigate porosities [15]. Other CT studies contribute with physical properties of sediment cores [16,17,18,19]. Several companies propose CT analyses to mining companies, in order to detect, e.g., gold on hidden tectonic structures [20].

Iron ore deposits, in particular banded iron formations, present alternating layers of iron oxide concentrates and silica-rich layers (quartz), while in some deposits iron oxide layers occur in alternation with carbonate layers [21]. These deposits may also host heavy minerals [22], e.g., Ba sulfate and oxides, rare earth elementphosphates (REE-phosphates) and U-oxides, which are harmful for mineral processing, metallurgical extraction and waste management. The layers and lenses have variable thicknesses (millimeters to meters thick). Deformation and/or metamorphism can lead to lens shaped or nodular iron formations at different scales [21,22].

Iron ore grades are important for the market value. Low grades, such as 57% of iron, are more and more frequent than higher-grade values >60% of iron. Lower grade ores are usually composed of magnetite, while high grade ores host mainly hematite [23,24]. Commonly magnetite deposits are transformed into hematite through deformation, hydrothermal and supergene processes [25,26]. Furthermore, deformation creates fracture systems crosscutting the iron ores, and gold can precipitate from mineralizing hydrothermal fluids [27,28]. All these processes lead to changes at regional and local scale of densities, iron oxide mineralogy and particle sizes. These factors are important for setting up comminution parameters (for crushing, grinding and milling) for the processing industries. Quick and rough estimates of iron oxide distribution and ratios of economic to barren material will help to evaluate and adapt such parameters in an early stage of exploration and during processing. Furthermore, quartz volumes need to be estimated and eliminated in the first steps of beneficiation, as the material is highly abrasive and destroys the grinding and milling equipment.

Automatized studies of volume properties on such ores will contribute to elucidate the processes of formation as a fundamental study, and from the industrial point of view help for a faster decision making when processing these ores in order to anticipate dysfunction.

In the frame of the above-mentioned goals, the objective of this study was to perform first experiments by DE- and ME-XRT and CT on samples from different textured iron formations. The samples were well studied for mineralogy and chemistry [22,29,30]. These data contribute to CT and XRT interpretation.

## 2. Materials and Methods

### 2.1. Sample Material

The studied sample blocks come from small iron deposits in the Takab complex (130 km south-west of Zanjan) in northwestern Iran, tectonically belonging to the Alpine-Himalaya orogenic system [31]. This deposit is classified being of volcano sedimentary origin [32,33]. However, recent mineralogical and geochemical analyses provide evidences for a more complex history implying hydrothermal and metamorphic processes [29].

**Figure 1:**
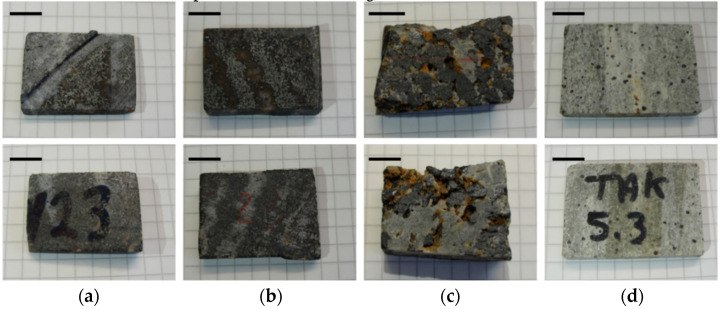
Front and back view of blocks analyzed by DE-, ME-XRT and CT: (**a**) banded iron oxide ore sample I (No. 23); (**b**) banded iron oxide ore sample II (No. 23); (**c**) nodular iron ore (No. 24); (**d**) silica-rich rock hosting disseminated iron oxide grains (No. 5.3). Scale bars are 10 mm. Samples from the Takab complex, NW Iran.

The iron oxide deposits are mainly hosted in metamorphic rocks such as mica schists and amphibolites. Four iron ore types are distinguished: (1) banded, (2) nodular, (3) massive and (4) disseminated iron ore. In this study, only banded, nodular and disseminated ores are analyzed to study the iron oxide concentrations in the light silica and/or carbonate-rich matrix. Samples can be seen in Figure 1.

The banded iron ore is characterized by discontinuous banding of iron oxide and quartz bands. Iron oxide filled veins crosscut the iron oxide and quartz bands. Magnetite ((FeO) Fe_2_O_3_) occurs as individual grains (~50 µm to several hundreds of µm). They include zircon (ZrSiO_4_), galena (PbS) and sphalerite (ZnS). Magnetite is partly transformed into hematite (Fe_2_O_3_). Goethite (FeOOH) is abundant around hematized magnetite and in veins. It sometimes hosts phosphates and pyrite (FeS_2_). The quartz matrix hosts also Mn–Ba oxides and barite (BaSO_4_). The latter replaces Ba–feldspar (hyalophane, (Ba,K)AlSi_3_O_8_). Rarely, uraninite (UO_2_) is found.

The nodular ore is characterized by iron oxide agglomerations of some millimeter in size. Other matrix minerals correspond to those in the banded ore. Magnetite includes phosphates, Mn-and Fe- carbonates ((Mn,Fe)CO_3_) and uraninite (UO_2_).

**Figure 2:**
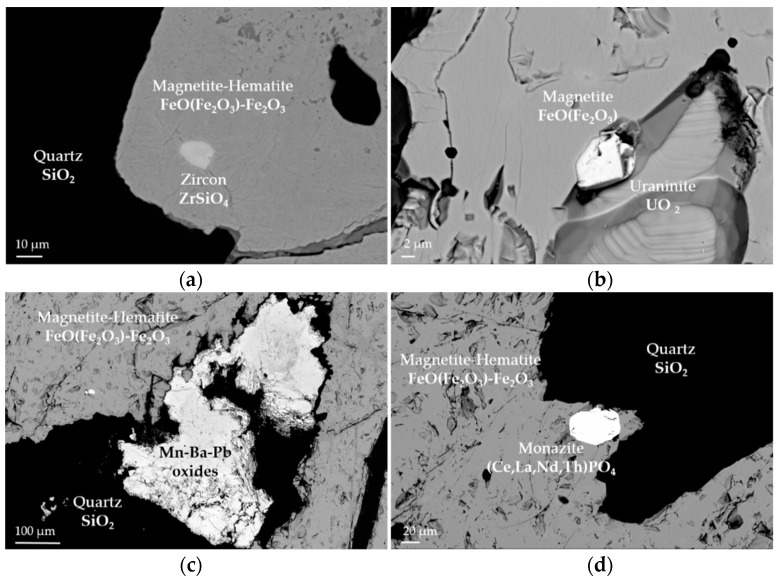
Images obtained by scanning electron microscope (SEM): (**a**) magnetite, partly transformed into hematite showing zircon inclusion; (**b**) magnetite with uraninite inclusion; (**c**) Mn–Ba oxide interstitial to hematitized magnetite; (**d**) monazite inclusion in hematitized magnetite.

In the silica-rich rocks, disseminated magnetite (~400 µm to 1.5 mm) is partly transformed into hematite. Goethite (FeOOH) surrounds partly the oxides. Oxides show uraninite (UO_2_), REE-phosphates, galena (PbS), sphalerite (ZnS) and zircon inclusions (Figure 2). In the silica matrix, minor K-feldspar (K(AlMg)_2_(OH)_2_(SiAl)_4_O_10_) and phengite, a hydrous silicate, rutile (TiO_2_), REE-phosphates, scheelite (CaWO_4_) and barite (BaSO_4_) occur. All these samples are described in detail in [22].

In addition to the block samples, a banded iron (BIF) drill core of about 30 cm length was examined. It comes from a deposit that belongs to the Transvaal Supergroup in South Africa. The sample shows a laminated texture due to alternating bands of hematite as well as chert layers [34,35].

### 2.2. Sample Chemistry

Chemical analyses of the block samples were performed by laboratory XRF and inductively coupled plasma mass spectrometry (ICP-MS) on powdered rock samples at the SARM-laboratory (Nancy, France) and are published in [29]. The results are shown in Table 1.

The banded ore hosts 54.6 wt % of total Fe_2_O_3_ and 0.56 wt % FeO, which is related to the iron oxides, and 43.7 wt % of SiO_2_, mainly related to quartz. Low contents of Al_2_O_3_ (0.17 wt %) and CO_2_ (0.14 wt %) are hosted in clay minerals and carbonates, respectively. Sulfur is low (0.02 wt %), indicating that mostly oxides are present. Environmentally harmful elements such as As, Cd, Cr, Pb, Th and U are below 100 ppm.

The nodular ore sample is higher in Fe_2_O_3_ (66.8 wt %) and FeO (16.7 wt %), thus has higher iron oxide contents than the banded iron ore sample. It has a lower SiO_2_ (quartz) content (30.4 wt %). Al_2_O_3_ (0.14 wt %), CO_2_ (0.3 wt %) and S (0.04 wt %) are similarly as in the banded iron ore sample. MnO (2 wt %) is incorporated in iron oxides and may occur as Mn–Ba oxides (Figure 2c). Barium is present at 1990 ppm and forms distinct phases, such as barite or Ba oxides. Pb and Zn are elevated at 1026 ppm and 936 ppm, respectively, and are found as micrometric inclusions of PbS and ZnS in iron oxides. All other traces are below 65 ppm.

The silica-rich rock with disseminated oxides is rich in SiO_2_ (quartz and silicates, 70 wt %). Total iron (Fe_2_O_3_) is 5.7 wt %, with 0.9 wt % FeO. Al_2_O_3_ reaches 13.8 wt %, 7.4 wt % K_2_O and 0.25 wt % Na_2_O indicating the presence of feldspars. Barium content is high (2450 ppm) and is partly included in feldspars, and forms distinct phases, such as barite. The presence of zircons is indicated by Zr contents of 250 ppm.

For the drill core, there is currently no laboratory chemical analysis available. The sample was analyzed by a portable XRF-analyzer (TFS NITON 3T, mining mode). Measurements were performed on 20 points (window diameter 3 mm) on iron oxide and silica rich bands (Table 2) at DMT, Essen, Germany. Iron contents and silica contents vary strongly as micrometric quartz and iron oxide occur in both types of bands respectively. Iron rich bands contain 22% to 58% iron, while silica rich layers contain 2% to 6.2% of iron. An average of the iron rich bands indicates 42.4% of iron, an average of iron and silica rich bands gives an average of 23% of iron.

### 2.3. X-ray Transmission Measurements

**Figure 3:**
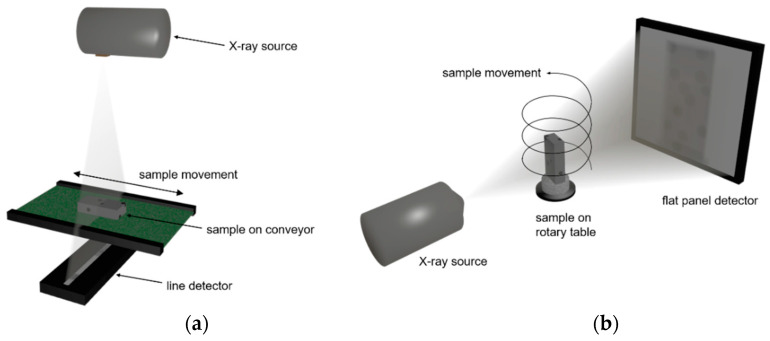
(**a**) Scheme of XRT system consisting of X-ray source, line detector and manipulator for moving the sample in between; (**b**) scheme of computed tomography (CT) system with X-ray source, flat panel detector and manipulator to rotate the sample between them. A simultaneous vertical translation of the sample results in a helical trajectory.

Both, DE- and ME-XRT measurements are usually performed by translating a sample between X-ray source and detector (typically a line-detector) using a conveyor belt or a drawer system (moving box on a linear stage, Figure 3a). While the sample moves through the X-ray beam path, the detector records an image line by line. This image is a projection of the sample’s X-ray attenuation properties into 2D space. Over the third dimension, i.e., along the X-ray beam path, the attenuation is integrated, leading to spatial averaging of the detected signal. The XRT systems used for the work presented in this paper were located at the Fraunhofer Development Center X-ray Technology (EZRT) in Fürth, Germany.

#### 2.3.1. Dual Energy X-ray Transmission

The DE-XRT measurements were performed using a system equipped with a DE line detector (DT X-Scan 0.8iL-410 DE-USB-C2) with 512 pixels and a pixel pitch of 0.8 mm. It consists of two detection layers. The first one and a 0.6 mm Cu filter act as prefilters for the second layer. In this way, the detector records simultaneously projections of the samples with two different X-ray energies. The used high-power X-ray source was a Comet MXR225/HP11. While the scans of the sample blocks were carried out with 220 kV and 0.9 mA, the drill core was measured with 2.2 mA and an additional prefilter of 0.5 mm Cu to account for the higher sample thickness. The samples were placed in a drawer that moved between source and detector at a speed of approximately 340 mm/s, corresponding to an exposure time of 2.67 ms per recorded line.

To evaluate the collected data, basis material decomposition (BMD) [4,36] was used. This method relies on the energy dependence of X-ray attenuation. When applied to two pure materials *l* (light) and *h* (heavy), it yields their respective areal densities *p_l_* and *p_h_*, i.e., their mass per area. From these areal densities, a virtual concentration can be calculated using *C_i_* = *p_i_*/(*p_l_* + *p_h_*), with *i* = *l* or *h*. However, the investigated samples are chemically complex compounds. Thus, their X-ray attenuation properties are characterized by an effective atomic number *Z*_eff_ calculated from the constituents’ atomic numbers *Z_i_* and partial chemical densities *ρ_i_* using $Z_{\mathrm{eff}}^{k}=\underset{i}{\sum }Z_{i}^{k}\rho_{i}/\underset{i}{\sum }\rho_{i}$, with *k* ≈ 3 [37]. Therefore, basis materials with an atomic number *Z* similar to the effective atomic number *Z*_eff_ of the materials of interest have to be chosen for the analysis. In this case, iron (*Z* = 26) and aluminum (*Z* = 13) were used, where the latter was chosen as many silicate rock types have *Z*_eff_ close to 13. The quantity obtained by BMD is consequently no true concentration, but a measure for the fraction of light and heavy materials.

#### 2.3.2. Multi Energy X-ray Transmission

In the ME-XRT setup, a conveyor belt system including a multi energy MultiX ME100 line detector was used (meanwhile sold by DT under the name X-Card ME3). The detector consists of three modules and has a total number of 384 pixels and a pixel pitch of 0.8 mm. The scanning width approximates to 307 mm. Each pixel has a single photon counter facilitating up to 128 energy channels in the range from 20 keV to 160 keV. The same type of source as for the DE measurements was used. Scans were carried out with 160 kV acceleration voltage and 0.5 mA tube current. A prefilter of 1 mm Ti was used to pre-harden the X-ray spectrum. The moving speed of the conveyor was approximately 40 mm/s, the exposure time 20 ms per recorded line.

The analysis is based on an algorithm described in [38,39]. It relies on a pixel wise calibration using combinations of aluminum and steel (X6Cr17) of different thicknesses. Similar to the BMD applied to DE-XRT measurements, this method yields no true concentration for the investigated samples, but the ratio of heavy iron-like and light aluminum-like materials.

While the requirement of extensive calibration measurements is a disadvantage compared to the established DE-XRT, a multi energy detector provides the possibility to adapt the energy binning to the specific application and thus to optimize the ability to distinguish materials and the quantitative accuracy of the results. For this paper, the energy channels were merged into two bins with energies below and above 112 keV, which gives good results for samples with high iron content. This flexibility is an advantage over a dual energy detector, where the difference between low and high energy channel is determined by its design.

### 2.4. Computed Tomography Scans

CT scans were performed at EZRT, Fürth, Germany. The X-ray inspection system (Figure 3b) used for the scans is composed of an Yxlon FXE-225.99(48) Microfocus X-ray source and a PerkinElmer XRD 1621 flat panel detector with CsI scintillator. The 2 × 2 pixel binning of the detector resulted in a pixel pitch of 200 µm.

While XRT methods only yield projections from one direction, during a CT scan, projections are recorded from different viewing angles. To this end, a helical trajectory was performed for all samples, meaning the sample was rotated between source and detector while simultaneously moving it in vertical direction (Figure 3b) [40]. In this way, 1200 projections per 360° rotation were acquired of the samples with 555 ms exposure time per projection. The tube voltage was 220 kV and 1 mm Cu was used as pre-filter to reduce beam-hardening artifacts.

**Figure 4:**
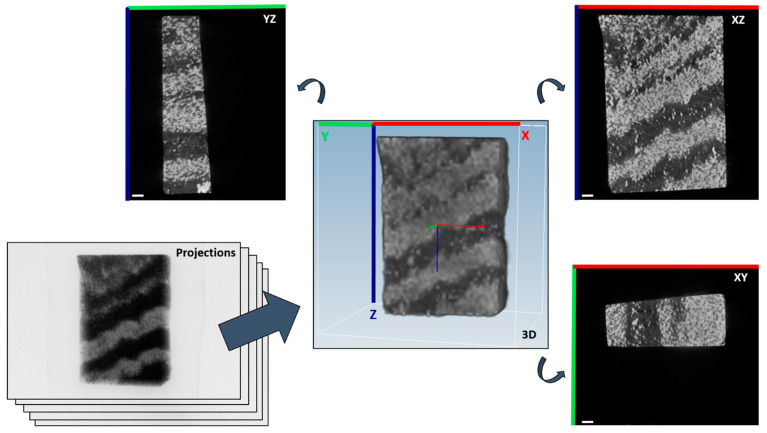
The reconstruction of the projection data with the filtered backprojection algorithm results in a 3D volume. Virtual cross sections through this volume can be created in any arbitrary direction and reveal, for instance, the internal orientation of bands. The shown sample is the banded iron oxide sample II in Figure 1a. Scale bars (bottom left) are 2 mm.

3D volume datasets were reconstructed from the X-ray projections using a state of the art filtered backprojection algorithm. The volume consists of small 3D volume elements called voxels (similar to the 2D picture elements called pixels) with an edge length of 36 µm. Virtual cross sections through the volume allow the examination of internal structures (see Figure 4).

The 3D volume datasets of the block samples were rotated so that the axes of the almost cuboid-like rocks are approximately aligned to a Cartesian coordinate system for better display.

### 2.5. Image Processing of CT Data

Characteristic information on the iron formations like the internal particle size, can be obtained from their respective 3D reconstructions [41]. As a first step, the data was filtered using a median filter with a 5 × 5 × 5 ball mask for noise reduction. This was followed by a so-called blob analysis. This image processing method is used to detect regions consisting of connected voxels that differ in distinct properties (here: grey values). These groups of voxels are called ‘binary large objects’ or blobs [42]. For this purpose, the 3D reconstructions are binarized using a threshold value, which was accomplished automatically by Otsu’s method [43]. In the emerging binary mask, objects can be segmented using a watershed transformation [44]. Thereby, information like blob size and volume can be calculated. Blobs less than or equal to five voxels were removed for the following analytical steps. The volume fraction *V*_Fe_ of iron rich regions per sample was obtained by summing the size of all detected blobs and dividing it by the total number of voxels per rock.

## 3. Results

### 3.1. Banded Ore

**Figure 5:**
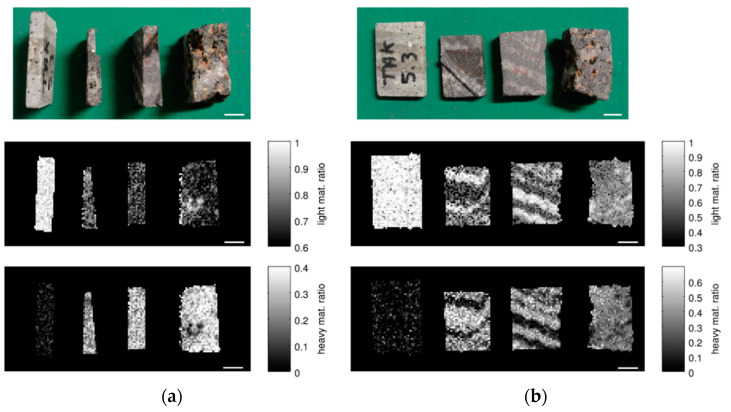
Multi energy X-ray transmission imaging (ME-XRT) examination of rock samples in (**a**) upright and (**b**) flat orientation. From left to right: silica-rich rock with disseminated oxides, banded iron ore I, banded iron ore II, nodular ore. While all samples appear in the image showing light material content *C_l_* (middle), the silica-rich rock with disseminated oxides hardly shows up in the heavy material content (*C_h_*) image (bottom). Layers in the banded iron ore samples are visible for the flat sample orientation (**b**) and are averaged out when turning the sample upright (**a**). Scale bars are 10 mm.

**Figure 6:**
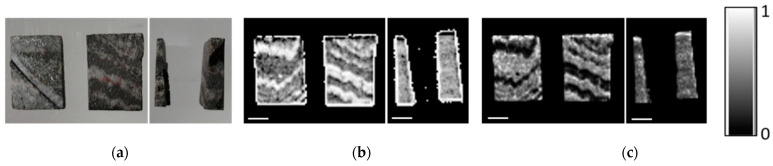
Dual energy X-ray transmission imaging (DE-XRT) examination of banded iron ore. Left rock is sample I, right rock is sample II: (**a**) photo of the samples in flat and upright orientation; (**b**) ratio of background material (Z_eff_ = 13) within the samples for both orientations; (**c**) ratio of heavy material (Z_eff_ = 26) within the samples for both orientations. Scale bars are 10 mm.

The optically visible bands are also revealed in DE- and ME-XRT measurements when choosing a suitable sample orientation (Figure 5 and Figure 6). Analysis shows that the visually darker regions have higher content of heavy materials (presumably iron). For sample orientations leading to averaging over areas with and without iron, the structural information is lost (Figure 5a). However, the ratios of heavy and light materials *C_h_* and *C_l_* can still be calculated.

Using DE-XRT, the content of heavy materials *C_h_* of samples I and II is 42% and 38% for the flat orientation and 38% and 39% for the upright orientation. The corresponding values for ME-XRT are 24% and 29% for the upright, and 37% and 32% for the flat orientation, respectively. The difference between the two orientations is caused by the limitation of the X-ray tube voltage to 160 kV in the ME-XRT setup. For the upright orientation, the transmission through the samples falls below 5% in iron rich regions, which leads to an underestimation of *C_h_* (see also Section 4, Discussion).

Analysis of CT data using blob analysis can also be used to determine the volume ratio of the parts with high iron content. While for sample I 9900 blobs bigger than five voxels were found, in sample II 18,739 blobs were identified (Table 3). In total, all blobs sum up to volume fractions of 39% and 41%. The ratios found for samples I and II are comparable to those found by DE-XRT.

**Figure 7:**
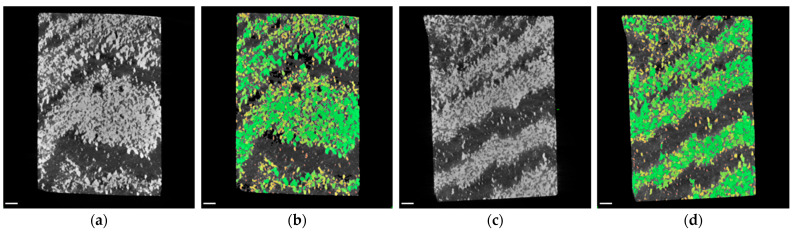
CT examination of banded iron ores: (**a**) virtual cross section through reconstruction in XZ‑plane of sample I; (**b**) results of blob analysis of sample I, identified blobs are labeled green, orange and yellow (colors are related to blob size); (**c**) virtual cross section through reconstruction in XZ‑plane of sample II; (**d**) results of blob analysis of sample II. Scale bars are 2 mm.

Exemplary virtual cross sections of both samples with and without blob analysis can be seen in Figure 7. The identified blobs are labeled in green, orange and yellow according to their size: green blobs are bigger, while dark orange indicates the smallest emerging blobs. By reason of the high contrast between the light background material and the heavy material-of-interest, the blob analysis worked very well.

**Figure 8:**
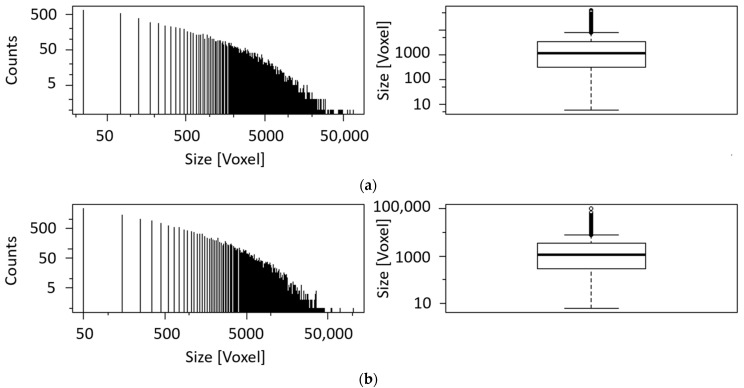
(**a**) Histogram and boxplot of the blob size of sample I. The black line in the middle of the box indicates the median (1234), while the black lines framing the box are the lower (324.8) and upper quartile (3439.2); (**b**) histogram and boxplot of the blob size of sample II. The black line in the middle of the box indicates the median (1135), while the black lines framing the box are the lower (296) and upper quartile (3426).

A histogram and a boxplot for the calculated blob size are shown in Figure 8. Due to the exclusion of blobs smaller than six voxels from the analysis, an apparent cut-off can be seen in the histogram at minimum size. The histogram is constructed by dividing the range of values into a series of equally wide intervals (‘bins’) and counting how many values fall into each of them. For reasons of clarity, the bins are represented by lines in the center of their respective interval. Boxplots are a tool to display a dataset based on its important percentiles: median (50th percentile), lower quartile (25th percentile), and upper quartile (75th percentile). Even though the number of blobs of sample II is the twofold of sample I, the boxplots of both samples are very similar with corresponding median and quartile values.

### 3.2. Nodular Ore

**Figure 9:**
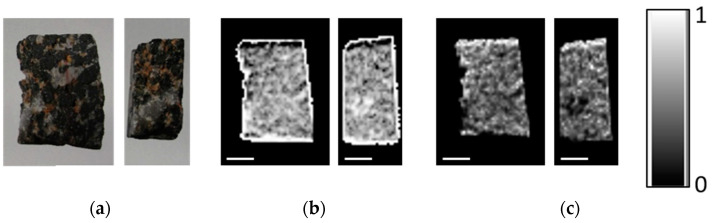
DE-XRT examination of nodular ore: (**a**) photo of the sample in flat and upright orientation; (**b**) ratio of background material (*Z*_eff_ = 13) within the sample for both orientations; (**c**) ratio of heavy material (*Z*_eff_ = 26) within the sample for both orientations. The scale bars are 10 mm.

As the nodules are distributed throughout the sample, they are averaged out in a transmission image and thus cannot be visualized clearly using XRT independent of the sample orientation (Figure 6 and Figure 9). The portion of heavy materials *C_h_* found by DE-XRT is 41% for the flat and 39% for the upright orientation, while ME-XRT analysis yields 32% and 31%, respectively.

**Figure 10:**
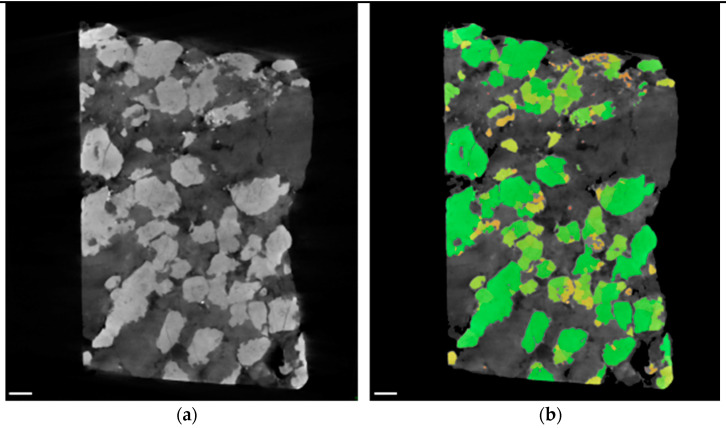
CT examination of nodular iron ore: (**a**) virtual cross section through reconstruction in XZ‑plane. Nodules (bright) and pores (black) are visible; (**b**) results of blob analysis, identified blobs are labelled green, orange and yellow (colors are related to blob size). Scale bars are 2 mm.

**Figure 11:**
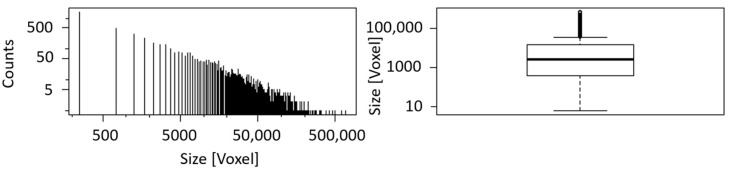
Histogram and boxplot of the blob size of nodular iron ore. The black line in the middle of the box indicates the median (2746), while the black lines framing the box are the lower (369) and upper quartile (14,941).

Blob analysis of CT data indicates a volume fraction of 40% of the iron rich phase while exhibiting 5557 blobs (see Figure 10 and Table 4). Boxplot and histogram of the blob size can be seen in Figure 11. The blob size found in nodular ore is much bigger in comparison to the blob size of the banded iron samples, while the number of blobs is much smaller, leading to a similar volume fraction around 40%.

**Figure 12:**
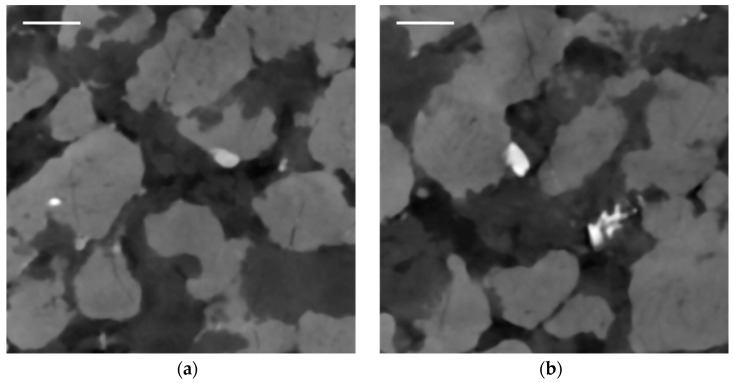
Virtual cross section through reconstruction in XZ-plane of nodular ore: (**a**) inclusions inside the nodules are visible; (**b**) inclusions inside the background material are visible. Scale bars are 2 mm.

Finally, a close look on the virtual cross section of the nodular ore sample reveals inclusions inside the nodules and the background material similar to the SEM examination in Figure 2 and [22]. Bright inclusions inside the magnetite-hematite nodules (Figure 12a) might be zircon, uraninite, monazite or Cu- and Ni-sulfides, while inclusions inside the background material (Figure 12b), presumably quartz, are probably Ba-sulfates or Mn–Ba oxides.

### 3.3. Silica-Rich Rock with Disseminated Iron Oxides

**Figure 13:**
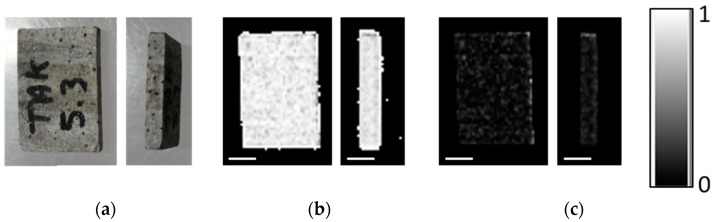
DE-XRT examination of silica-rich rock with disseminated iron oxides: (**a**) photo of the sample in flat and upright orientation; (**b**) ratio of background material (*Z*_eff_ = 13) within the sample for both orientations; (**c**) ratio of heavy material (*Z*_eff_ = 26) within the sample for both orientations. The scale bars are 10 mm.

The silica rock with disseminated iron oxides shows the lowest iron content of the investigated samples, which can clearly be seen in Figure 13c. The values for *C_h_* found using DE-XRT are 12% (upright) and 8% (flat), and for ME-XRT 4% (upright) and 6% (flat).

**Figure 14:**
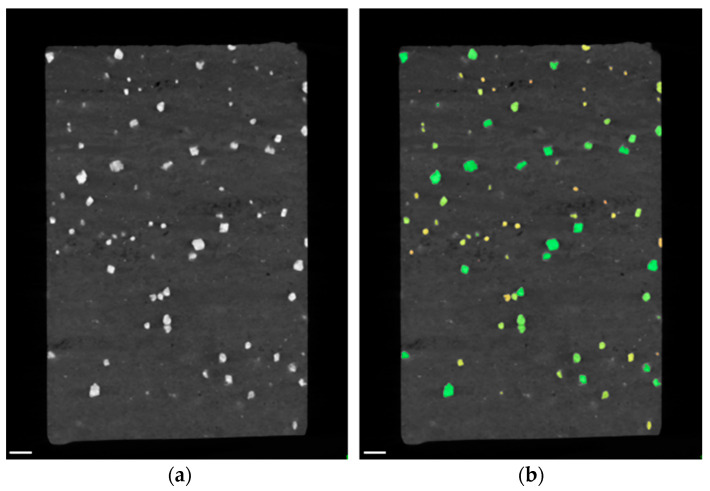
CT examination of silica-rich rock with disseminated iron oxides: (**a**) virtual cross section through reconstruction in XZ-plane; (**b**) results of blob analysis, identified blobs are labelled green, orange and yellow (colors are related to blob size). Scale bars are 2 mm.

Blob analysis of the CT data revealed 2% volume fraction of the iron rich phase, possessing only 847 blobs (Table 5). While the small iron-rich particles are barely visible using XRT due to averaging over one dimension and the lower spatial resolution (Figure 5 and Figure 13), they are clearly revealed by CT and can easily be found with the blob analysis (Figure 14).

**Figure 15:**
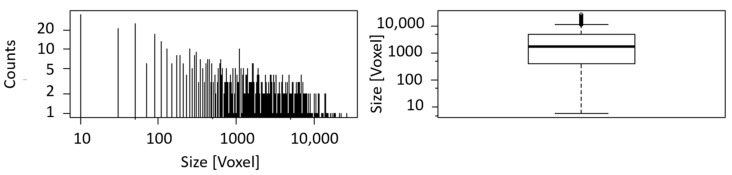
Histogram and boxplot of the blob size of nodular iron ore. The black line in the middle of the box indicates the median (1747), while the black lines framing the box are the lower (409) and upper quartile (4778).

Although the sample of silica rich rock with disseminated iron oxides exhibits rock size in voxels and boxplot attributes (Figure 15) similar to the banded iron ore sample II, they differ tremendously in the number of blobs leading to the volume fraction of the iron rich phase (2% and 41%, respectively).

### 3.4. Drill Core

**Figure 16:**
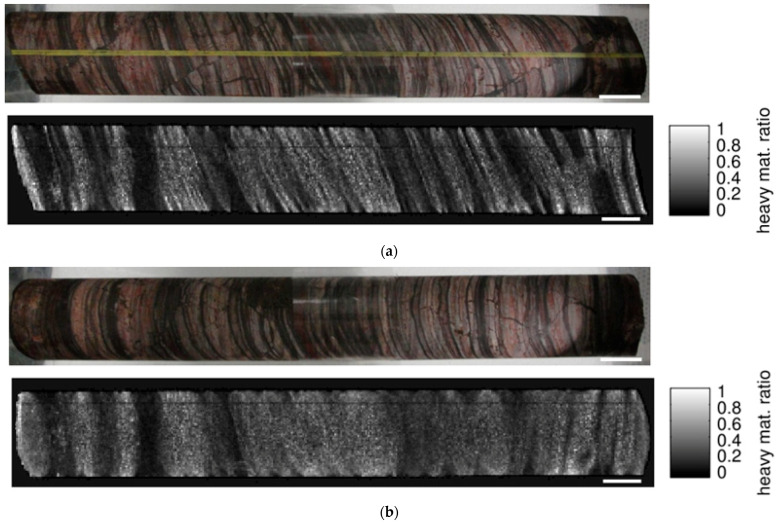
Photograph and DE-XRT analysis of the banded iron drill core: (**a**) the layering of the sample is revealed for suitable orientation; (**b**) after rotation of the core by 90° the structure is obscured. Scale bars are 20 mm.

Due to its thickness and the resulting high absorption in the iron rich phases, the drill core could not be analyzed using ME-XRT, which is limited to 160 kV due to the used detector. Using DE-XRT, the layer structure of the sample is clearly visible for one orientation, but blurred when turning the sample by 90° (Figure 16). The content of heavy materials was found to be 17% for the first and 20% for the second orientation.

**Figure 17:**
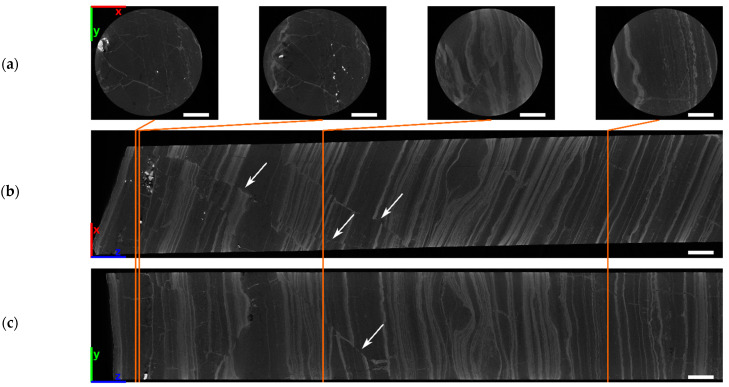
Virtual cross sections along different directions through the reconstructed CT volume of the banded iron drill core: (**a**) XY-plane; (**b**) XZ-plane; (**c**) YZ-plane. Highly absorbing inclusions (bright spots) are visible at the left end of (**b**) and (**c**). The left part also reveals microfaults (white arrows). Scale bars are 10 mm.

A CT scan was performed for 25 cm of the core sample. Virtual cross sections (Figure 17) through the volume reveal different structural features. The width and frequency of the layers varies along the length of the core. Additionally, microfaults are visible and heavy element inclusions are revealed as bright spots on the left end of Figure 17b,c and inside two virtual cross sections of Figure 17a.

**Figure 18:**
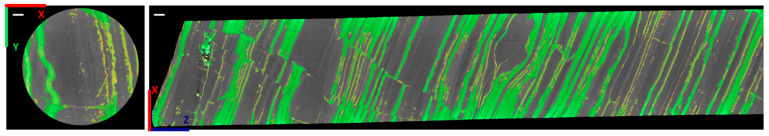
Virtual cross sections along different directions through the reconstructed CT volume of the banded iron drill core after blob analysis. Identified blobs are labelled green, orange and yellow (colors are related to blob size). Orientation was chosen to be the same as in Figure 17a,b. Scale bars are 2 mm.

**Figure 19:**
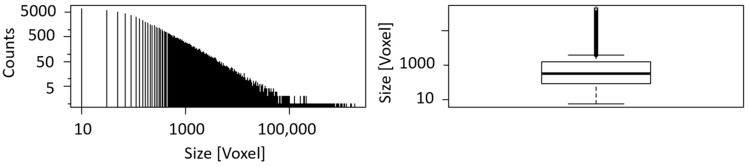
Histogram and boxplot of the blob size of the banded iron drill core. The black line in the middle of the box indicates the median (257), while the black lines framing the box are the lower (76) and upper quartile (1110).

The blob analysis of the banded iron drill core found 91,649 blobs (Table 6 and Figure 18), corresponding to a volume fraction of *V*_Fe_ = 40 %. Due to the low contrast between the iron-rich bands and the background material, the threshold could not be achieved by using Otsu’s method. Therefore, an appropriate threshold was chosen manually. Unlike the previous samples, the drill core does not contain pure iron oxide and quartz layers. However, in combination with the iron concentration of the iron rich and iron poor phases $C_{\mathrm{Fe}}$ and $C_{\mathrm{Fe}}^{*}$ obtained by XRF (Table 2), the iron content can be estimated. Using $V_{\mathrm{Fe}}C_{\mathrm{Fe}}+\left(1-V_{\mathrm{Fe}}\right)C_{\mathrm{Fe}}^{*}=0.4\times 0.424+0.6\times 0.0358$ an iron content of approximately 19% is calculated. Boxplot and histogram of the blob size can be seen in Figure 19.

## 4. Discussion

Table 7 shows a comparison of the content of heavy materials obtained by the different methods. In this comparison, it has to be considered that all of them measure different quantities. The element sensitive chemical composition obtained by XRF and ICP-MS can be considered as ground truth. However, it has to be kept in mind that ICP-MS requires the destruction of the sample. The chemical analyses were thus not performed on the samples studied by CT, DE- and ME-XRT, but on their counterparts created when cutting them from the rock. Therefore, they should be taken as indicative. Neither XRT nor CT give the ‘true’ iron content, but quantities from which it can be derived. However, these methods could be calibrated using wet chemical analysis if necessary. In this case, laboratory chemical studies should be performed on the same samples as analyzed by XRT and CT.

The XRT methods give the fraction of heavy materials. It equals the iron content only under the assumption that the samples consist only of iron and aluminum, which was used as basis material as silica rocks have a similar effective atomic number. Given the chemical composition of the investigated samples (Table 1), this is justified. However, it might not be valid for all kinds of rocks and should thus be kept in mind when interpreting DE- or ME-XRT data. The disagreement between the two XRT methods for the banded and nodular ore blocks is related to the different tube voltages and the high fraction of heavy materials. Experience shows that high contents of heavy materials tend to be underestimated by ME-XRT for samples differing from the calibration materials. For instance, using a tube voltage of 160 kV and steel as calibration material, underestimation is found in the analysis of measurements of more than 5 mm of pure iron. A higher tube voltage would increase the transmission and result in a better accuracy for high fractions of heavy materials. Unfortunately, this is not possible with the currently used detector. However, for sufficient transmission, the found fraction of heavy materials is proportional to the true concentration.

The blob analysis of CT data reveals the fraction of the iron rich phase by its elevated attenuation, but not the iron content itself. For the block samples, these two values can be equated with each other due to their chemical composition. However, this is not the case for the measurements on the drill core presented in Section 3.4. This banded iron ore hosts red jaspilite layers. These layers consist of a mixture of quartz hosting multiple nano- and micro-sized dispersed iron oxides. If assuming pure quartz and iron oxide layers for data interpretation, erroneous results are obtained. Thus, the conversion of the volume fractions of iron and quartz rich bands to the iron content must be calibrated with portable XRF and laboratory chemical analyses. With this combination of CT and chemical data shown in Section 3.4, the iron content can be obtained and agrees well with the values from the other methods.

The data in Table 7 show clearly, that iron rich and iron poor samples can be distinguished using DE-XRT, ME-XRT and CT. Smaller variations in iron rich samples seem to be erroneous. For analyses on characteristic samples in an exploration campaign, the here applied methods allow a rapid access on the rough contents of heavy and very heavy materials.

Apart from the chemical composition of a rock, also structural information is of interest. XRT is inherently connected to spatial averaging due to integration of the detected signal along the X-ray beam path. Thin samples (in comparison to the internal structures) might still reveal some of their structure. However, for thick samples this might not be the case for instance due to disadvantageous orientation as seen in the cases of banded or nodular iron ores. Here, CT is clearly beneficial as it allows to examine arbitrary virtual cross sections of a sample. This allows seeing the spatial distribution of bands, nodules and pores. The orientation of layers can also be estimated, when suitable GPS data are available. In addition, a reconstructed CT volume can reveal the presence of additional materials that differ in their X-ray attenuation.

## 5. Conclusions

Both, XRT and CT, reveal interesting information regarding the composition of the investigated samples. While the measured values of the iron content vary depending on the applied method, they show the same trend, i.e., the rock with disseminated iron oxides shows by far the lowest (~4%), the other block samples the highest content (~40%) of all investigated samples and the BIF drill core, although it resembles the banded iron block samples in appearance, has a medium iron content (~20%). The latter can be explained by the different origins of the samples: the BIF drill core originates from South Africa and the smaller samples from Iran.

CT allows to obtain structural information to which XRT methods have only limited access. While ordinary CT is useful to relate the volume of iron-rich phases to those with low iron content, it would also be possible to perform dual energy CT. This could allow to determine the iron content of the rich phases in a way similar to DE-XRT.

A major difference between XRT methods and CT is the amount of time needed for a measurement. While XRT can be used as a real-time inline process, the time for recording and reconstruction of CT data depends strongly on the acquisition parameters and is typically in the range of hours per meter. Still, in comparison to wet chemical analysis, which above all requires destruction of the sample, this might save time. Therefore, depending on the required information, a suitable method should be chosen. For instance, in exploration XRT could be used for continuous monitoring, while CT would be performed additionally for selected samples in a workshop at mine site or laboratory if there are indications for changes in the geologic situation. The machine and the measurement protocol, X-ray parameters, resolution and so on, can be adapted to the deposit and the questions to be answered. Thus, a custom solution is possible for different mining sites.

## Figures and Tables

**Table 1:** Major, minor and trace element composition of the three iron oxide bearing rocks: banded iron ore (No. 23), nodular iron ore (No. 24) and silica-rich rock containing disseminated iron oxides (No. 5.3) (analyses: CRPG SARM, [29]).

Elemental Composition	Unit	Banded Ore	Nodular Ore	Silica Rich Rock + Iron Oxides
SiO_2_	wt %	43.7	30.4	70.1
Al_2_O_3_	wt %	0.17	0.14	13.72
Fe_2_O_3_ (total)	wt %	54.58	66.77	5.73
MnO	wt %	0.05	2.01	<D.L. ^1^
MgO	wt %	<D.L.	<D.L.	0.58
CaO	wt %	0.06	0.14	0.05
Na_2_O	wt %	<D.L.	<D.L.	0.25
K_2_O	wt %	<D.L.	<D.L.	7.64
TiO_2_	wt %	<D.L.	<D.L.	0.42
P_2_O_5_	wt %	0.12	<D.L.	<D.L.
LOI ^2^	wt %	0.8	−1.390	1.54
Total	wt %	99.45	98.11	100.04
CO_2_ (total)	wt %	0.14	0.30	0.17
FeO	wt %	0.56	16.69	0.88
S (total)	wt %	0.02	0.04	<0.01
As	ppm	19.8	8.3	30.5
Ba	ppm	11.6	1991	2448
Cd	ppm	<D.L.	59.5	0.2
Co	ppm	5.2	1.2	<D.L.
Cr	ppm	8.8	7.8	18.9
Mo	ppm	11.6	5.4	<D.L.
Nb	ppm	0.1	0.25	10.3
Ni	ppm	<D.L.	<D.L.	<D.L.
Pb	ppm	4.8	1026	19.5
Th	ppm	0.5	0.1	16
U	ppm	0.8	1.8	1.9
V	ppm	84	64	39
W	ppm	<D.L.	<D.L.	38
Zn	ppm	24	936	16
Zr	ppm	6	3	250
Fe (total)	wt %	38.2	46.7	4
Fe ^2 3^	wt %	0.44	12.97	0.7

^1^ Detection limit; ^2^ loss on ignition; ^3^ calculated from FeO.

**Table 2:** Results of XRF measurements on 20 points (10 each for iron rich bands and silica rich layers) of the banded iron (BIF) drill core sample.

Silica Rich Layers			Iron Rich Bands		
Point	Si wt. %	Fe wt. %	Point	Si wt. %	Fe wt. %
1	48.3	5.2	1	23.1	42.3
2	52.7	4.2	2	7.9	41.9
3	54.0	3.1	3	29.0	40.4
4	50.7	2.6	4	23.5	32.1
5	53.1	6.2	5	38.2	21.8
6	55.1	2.0	6	6.2	53.8
7	48.1	4.6	7	9.2	44.3
8	55.3	2.3	8	23.9	45.3
9	54.3	2.4	9	8.0	58.0
10	51.9	3.2	10	17.3	44.1
Average	52.35	3.58	Average	18.63	42.4

**Table 3:** Results of the blob analysis for banded iron ore I and II.

Sample	Number of Blobs	Number of Blobs (≤5 Voxels)	Sum of Blob Size (Voxels)	Rock Size (Voxels)	Volume Fraction
I	10,107	9900	27,425,752	70,544,776	39%
II	19,136	18,739	52,684,052	129,794,691	41%

**Table 4:** Results of the blob analysis for nodular ore.

Number of Blobs	Number of Blobs (≤5 Voxels)	Sum of Blob Size (Voxels)	Rock Size (Voxels)	Volume Fraction
5697	5557	91,319,546	227,064,431	40%

**Table 5:** Results of the blob analysis for silica-rich rock with disseminated iron oxides.

Number of Blobs	Number of Blobs (≤5 Voxels)	Sum of Blob Size (Voxels)	Rock Size (Voxels)	Volume Fraction
872	847	2,671,441	140,002,072	2%

**Table 6:** Results of the blob analysis for the banded iron drill core.

Number of Blobs	Number of Blobs (≤5 Voxels)	Sum of Blob Size (Voxels)	Rock Size (Voxels)	Volume Fraction
101,595	91,649	349,083,989	882,009,034	40%

**Table 7:** Comparison of results obtained by different methods. Chemical analysis as the ground truth gives iron content, DE- and ME-XRT give the ratio of heavy materials, CT gives the volume fraction of the iron-rich phase.

Sample	Chemistry	DE-XRT (Upright)	DE-XRT (Flat)	ME-XRT (Upright)	ME-XRT (Flat)	CT
Disseminated	4%	12%	8%	4%	6%	2%
Banded I	38.2%	38%	42%	24%	37%	39%
Banded II	38.2%	39%	38%	29%	32%	41%
Nodular	46.7%	39%	41%	31%	32%	40%
Drill core	23% ^1^	20%	17%	n.a. ^2^	n.a.	40% ^3^, 19% ^4^

^1^ XRF analysis; ^2^ not analyzed; ^3^ volume fraction of iron rich bands; ^4^ estimated iron content.

## Data Availability

Not applicable.
